# Determining the Best Approach: Comparing and Contrasting the Impact of Different Coping Strategies on Work-Related Stress and Burnout Among Saudi Commercial Pilots

**DOI:** 10.7759/cureus.41948

**Published:** 2023-07-16

**Authors:** Abdullmajeed A Alghamdi, ‏Amal H Alghamdi

**Affiliations:** 1 Medical Directorate, Saudi Royal Land Forces, Riyadh, SAU; 2 Preventive Medicine Postgraduate Program, Ministry of Health, Jeddah, SAU

**Keywords:** cross-sectional studies, working hours, oldenburg inventory, public health, occupational health, mental health, community medicine, preventive medicine, aviation medicine

## Abstract

Background

Many physiological, psychological, and environmental stressors can be experienced by pilots throughout their careers, which may affect their mental and psychological health and their performance consequently. Stressing factors of aviation and commercial operations can result in pilots’ burnout, which is a description of the response to chronic emotional exhaustion and loss of motivation.

Objective

The objective of this study was to determine the prevalence of burnout and compare the effects of different coping strategies on burnout levels among commercial pilots in the Kingdom of Saudi Arabia, 2023

Methods

The study was a cross-sectional survey utilizing an online form of a validated questionnaire administered to pilots of Saudi commercial airlines in the Kingdom of Saudi Arabia. The study targeted on-duty Saudi commercial pilots, excluding retired and in-training pilots, with an estimated population of around 2000. A sample size of 311 was determined using an equation considering a 5% margin of error, 95% confidence level, and a 60.3% burnout prevalence rate. Non-probability snowball sampling method was used to achieve sample size.

Results

A total of 321 pilots were included, mostly Saudis. The participants had a median age of 47, and the majority were married with one to two children. BMI classifications showed high percentages of overweight and obese individuals. The study found that most pilots experienced medium levels of burnout (70.1%). Nationality, marital status, and shorter sleeping duration were significantly associated with higher burnout levels. Common challenges included long duties (79.4%), irregular working hours (72.3%), and time away from home (55.5%). Coping strategies included rest and sleep (81%), exercise (59.2%), and relaxation behaviors (50.5%). Stressors such as long duties, irregular working hours, and work conflicts were significantly associated with higher burnout levels. However, no significant associations were found between coping strategies and burnout levels.

Conclusion

The study found that most pilots experienced medium levels of burnout. In addition, common challenges included long duties, irregular working hours, and time away from home. The study emphasizes the need to address work-related stressors, implement interventions, and support pilots' mental health. Promoting healthy coping strategies and understanding individual resilience is important. Further research and organizational efforts are required to mitigate burnout and enhance the quality of life for aviation professionals, benefiting both individuals and the industry.

## Introduction

Pilots encounter various physiological, psychological, and environmental stressors throughout their careers, which can have an impact on their mental and psychological well-being as well as their performance [1]. The nature of their work exposes them to multiple stressors, including manually controlling critical flight phases, operating complex aircraft systems, interacting with crew members and air traffic control, and handling emergency situations [2]. Additionally, factors such as working in rotational shifts, long hours, and the stressful environment of confined cabin space, along with exposure to noise and light, contribute to the stress experienced by pilots in aviation and commercial operations. These stressors can lead to burnout among pilots, characterized by chronic emotional exhaustion and a loss of motivation [3].

Work-related stress (WRS) refers to the imbalance between the demands of work and the abilities and knowledge of the workers. It encompasses various factors such as job stress, employment conditions, concerns about job security, and the struggle to maintain a work-life balance [4]. For pilots, these stressors are particularly critical as they must be in optimal physical and psychological health to ensure the safety of flights and aviation systems. While previous studies on pilots have primarily focused on issues like fatigue and insomnia, there is a growing recognition of the importance of mental and psychological well-being [5-7]. Burnout and depression have been identified as significant concerns in pilots' mental health [5,8]. The inclusion of burnout as a syndrome in the WHO's International Classification of Diseases 11th Revision (ICD-11) in 2019 further emphasizes its significance as a health issue across various professional domains [9]. A previously published study highlighted a high prevalence of burnout, with (32.6%) of the study population affected [10]. The presence of burnout can lead to an increased likelihood of errors and a diminished prioritization of safety [11]. Recognizing the importance of mental health, the European Aviation Safety Authority (EASA) has introduced new rules concerning mental health issues in pilots [12]. Additionally, there is a growing emphasis on promoting well-being and adopting healthy practices among aviation professionals [13].

The Maslach Burnout Inventory (MBI) [14] is indeed one of the oldest and most well-known instruments for measuring burnout. However, there are also other valid options available that can be used to measure burnout in any occupational group, and some of these options are better suited to the conceptualization of burnout as a two-factor construct. Examples of alternative burnout measuring scales include the Bergen Burnout Inventory (BBI) [15], Copenhagen Burnout Inventory (CBI) [16], and Oldenburg Burnout Inventory (OLBI) [17]. These scales have been developed and validated to assess burnout in different contexts and populations. The OLBI, in particular, has been applied to various worker groups, including healthcare professionals [18], teachers [19], executive directors [20], and airline pilots [3], demonstrating its versatility and reliability. Studies using the OLBI have generally reported good values of internal consistency, indicating its reliability as an instrument for assessing burnout across different labor contexts. The diversity of occupational groups in which the OLBI has been successfully applied strengthens its credibility and suggests its suitability for use in different work settings.

In a previously published study, researchers aimed to examine the work characteristics and outcomes related to burnout among pilots. The study involved an online survey administered to 1147 pilots who were members of the European pilots professional association. To measure burnout, the researchers utilized OLBI. The OLBI offers advantages over other validated burnout tools as it includes both positive and negative items, aligning with psychometric standards. Additionally, the OLBI has been found to be a better predictor of long-term health compared to depression or anxiety alone, making it suitable for use among pilots. Using specific cut-off scores, the study revealed that 40% of the pilots had very high burnout scores, while 20.3% had high burnout scores. Furthermore, 88% of the participants reported frequently observing their colleagues already fatigued when starting their working shifts. The study suggests the implementation of interventions such as burnout diagnosis and support, the redesign of workloads, and facilitating the approval of requested off-duty days to positively impact pilots' performance [3].

A qualitative study was conducted to explore the relationship between WRS and the well-being and performance of commercial pilots. Thirty-three pilots participated in three workshops aimed at validating and mapping the impact of WRS on pilots' well-being, as well as validating the findings regarding the association between WRS and well-being and performance. The study revealed that the participants were initially unaware of mental health (MH) issues and that MH issues were not identified or addressed. The pilots reported experiencing major WRS factors such as fatigue, working in high-altitude environments, sleep disturbances, lack of breaks, social isolation, a sedentary job environment, and inadequate support from flight operations and management. The study concluded that pilots need to receive training in coping strategies and risk identification to ensure their mental health, as it is an integral part of overall health that requires effective management [1].

Another study was conducted to explore the relationship between WRS, its effects on pilots' well-being, and the utilization of coping strategies. A web-based survey was administered to 1059 pilots, which included measures for depression by Patient Health Questionnaire-9 (PHQ-9) and burnout (OLBI). The study found that a majority of pilots (83.4%) agreed that WRS had an impact on their performance. Sleep difficulties and musculoskeletal symptoms were reported as the most common negative impacts on well-being (81% and 73.5%, respectively), with irregular working hours being the most frequently reported source of WRS. In terms of depression prevalence, 40% exhibited mild clinical depression. The majority of pilots (59.3%) reported using coping strategies, which were found to have a positive impact on depression severity. Common coping strategies included regular exercise, sufficient rest and sleep, a healthy diet, relaxation techniques, seeking professional support, engaging in conversations with colleagues, and maintaining connections with family and friends. In conclusion, coping strategies were found to have a positive effect on pilots' mental health and well-being, contributing to a reduction in depression severity [21]. Therefore, this study aims to enhance the overall well-being and quality of life of pilots.

## Materials and methods

Study setting

The study was a cross-sectional survey utilizing an online form of a validated questionnaire administered to pilots of Saudi commercial airlines in the Kingdom of Saudi Arabia.

Study population

The study focused on on-duty, active pilots employed by Saudi commercial airlines, excluding retired and in-training pilots. The estimated reference population of pilots was around 2000. To determine the sample size, the study used an equation considering a 5% margin of error, 95% confidence level (CI), and a prevalence rate of burnout among pilots at 60.3% [3]. The calculated sample size was 311. To reach this sample size, a non-probability snowball sampling method was employed.

Data collection

The questionnaire consisted of five sections. The first section included a welcome message, an overview of the study's objectives, and informed consent which was a mandatory question in order to start the questionnaire. The second section comprised questions related to participants' demographic characteristics. In the third section, participants were asked about various work-related stressors and their perceived mental health rating. The fourth section included questions based on OLBI [17]. Lastly, participants were asked about their most commonly used coping strategies, which were selected based on an extensive review of existing literature [13,21]. To introduce the online form to the pilots, initial contact was made through phone calls. During these calls, the researchers explained the eligibility criteria, the target population, and the nature of the sampling method to the potential respondents. The initial participants were then requested to share the link to the electronic questionnaire with their colleagues. To test the reliability of the OLBI tool, its Cronbach's alpha coefficient was calculated. The obtained score for reliability was α=0.83, indicating a high level of consistency and dependability in the results obtained from this tool. Data collection took place from January to April 2023.

Statistical analysis

The Statistical Package for the Social Sciences (IBM Corp. Released 2020. IBM SPSS Statistics for Windows, Version 29.0. Armonk, NY: IBM Corp) was utilized to perform the statistical analyses. To determine the normality of the distribution for continuous variables, the Shapiro-Wilk test was conducted. Continuous variables that followed a normal distribution were summarized using mean and standard deviation (SD), while those with a skewed distribution were summarized using median and interquartile range (IQR). Categorical variables were summarized using frequency tables and proportions. The Pearson Chi-Square test was used to investigate significant associations between categorical variables, while Spearman's correlation coefficient was utilized to examine correlations between continuous variables. The total burnout levels were classified based on mean and SD as follows: medium was defined as mean ±"1" SD, low as <"1" SD, and high as >"1" SD [22]. P-values less than 0.05 were considered statistically significant.

Ethical considerations

The research study obtained approval from the research committee at the Saudi Board of Preventive Medicine. Additionally, ethical approval was obtained from the Institutional Review Board of the Directorate of Health Affairs in Jeddah, the Kingdom of Saudi Arabia. Participants were duly informed about the nature and purpose of the study. Confidentiality of the data was ensured, and it was strictly used for research purposes only.

## Results

A total of 321 participants were included in the study, with the majority (97.5%) being Saudis. The median age was 47 years, with IQR 31-54. The median sleeping hours was 7 hours, with IQR 6-7. The participant’s body mass index (BMI) was further classified. While none of the participants were underweight, other classifications of normal, overweight, and obese were 27.4%, 44.2%, and 28.3%, respectively. The majority of the participants (76.9%) were married, 20.6% were single, and 2.5% were divorced. The number of children was one to two children among 28.0%, three to five children among 18.1%, and more than five children among 39.3% of the participants. Monthly income was categorized into five groups of 10,000 SAR (Saudi Arabian Riyal) intervals, with >40,000 SAR being the most frequently reported monthly income among 45.8% of the participants. 

**Figure 1:**
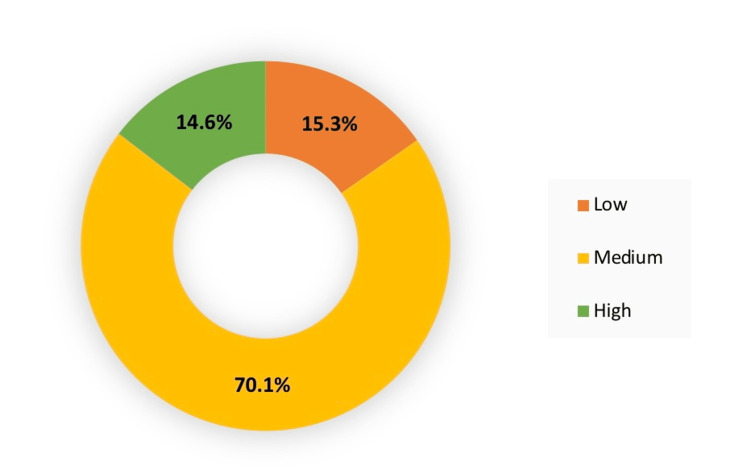
OLBI levels of burnout among pilots of Saudi commercial airlines OLBI: Oldenburg Burnout Inventory

The components of OLBI were computed to calculate the total for disengagement and exhaustion subscales and the total OLBI burnout level. The results showed that the disengagement median was 20 IQR (17-21), and the exhaustion median was 21 IQR (19-23). While the mean for total burnout scores was 40.39 ± 6.23. The total OLBI scores were further categorized according to standard deviations, the majority of pilots had a medium level of burnout (70.1%). The OLBI levels of burnout among pilots are demonstrated in Figure 1.

The statistical significance of the relationships between socio-demographic variables and levels of burnout was tested using the Chi-square test. Results showed significant associations of burnout level with nationality (p=0.032) and marital status (p=0.031). Also, shorter sleeping duration was found to be significantly correlated with higher burnout levels (p=0.015). A detailed description of the socio-demographic characteristics and their association with burnout level is shown in Table 1. 

**Table 1:** Socio-demographic characteristics and their association with burnout levels among pilots of Saudi commercial airlines SAR: Saudi Arabian Riyal

N=321	Total (%)	Burnout levels			P-value
		Low	Medium	High	
Nationality					
Saudi	313 (97.5%)	14.4%	70.6%	15%	0.032
Non-Saudi	8 (2.5%)	50%	50%	0%	
BMI					
Normal	88 (27.4%)	10.2%	71.6%	18.2%	0.319
Overweight	142 (44.3%)	14.8%	71.1%	14.1%	
Obese	91 (28.3%)	20.9%	67%	12.1%	
Marital status					
Married	247 (76.9%)	15.2%	59.1%	25.8%	0.031
Single	66 (20.6%)	15.8%	72.9%	11.3%	
Divorced	8 (2.5%)	0%	75%	25%	
Number of children					
None	90 (28%)	13.3%	65.6%	21.1%	0.56
1-2	58 (18.1%)	13.8%	72.4%	13.8%	
3-5	126 (39.3%)	17.5%	70.6%	11.9%	
>5	47 (14.6%)	14.9%	74.5%	10.6%	
Monthly income					
< 10K SAR	26 (8.1%)	26.9%	73.1%	0%	0.005
10K - 19K SAR	35 (10.9%)	11.4%	57.1%	31.4%	
20K – 29K SAR	79 (24.6%)	11.4%	70.9%	17.7%	
30K – 39K SAR	34 (10.6%)	5.9%	88.2%	5.9%	
> 40K SAR	147 (45.8%)	18.4%	68%	13.6%	
Smoking status					
Smoker	162 (50.5%)	12.3%	71%	16.7%	0.254
Non-smoker	159 (49.5%)	18.2%	69.2%	12.6%	

Respondents have reported experiencing various challenges and coping strategies in their jobs. The most commonly reported challenges were long duties (79.40%), irregular working hours (72.30%), and time away from home (55.50%). The most commonly reported coping strategies were rest and sleep (81.00%), exercise (59.20%), and relaxation behaviors (50.50%). See the full description of challenges and coping strategies in (Table 2). 

**Table 2:** Proportions of work-related stressors and coping strategies reported by pilots of Saudi commercial airlines

	N	%
Work-related stressors		
Long duties	255	79.4%
Irregular working hours	232	72.3%
Time away from home	178	55.5%
Anti-social hours	152	47.4%
Inflexible annual leave	151	47%
Work conflicts	144	44.9%
Unhealthy diet	119	37.1%
Miscommunication at work	119	37.1%
Changing nature of the industry	78	24.3%
Sedentary nature of the job	73	22.7%
Coping strategies		
Rest and sleep	260	81%
Exercise	190	59.2%
Relaxation behaviors	162	50.5%
Talking to family	143	44.5%
Healthy diet	112	34.9%
Talking to colleagues	103	32.1%
Professional support	84	26.2%
Other	49	15.3%

Further analyses included the stressors and their association with burnout levels. The stressors that were significantly associated with burnout (p<0.05) are long duties, irregular working hours, time away from home, inflexible annual leave, work conflicts, miscommunication at work, changing nature of the industry, and the sedentary nature of the job. Coping strategies were further analyzed for associations with burnout levels which did not show any statistical significance (p>0.05). See the detailed percentages of burnout levels among reported stressors in Table 3. 

**Table 3:** Association between burnout levels and work-related stressors among pilots of Saudi commercial airlines

Work-related stressors		Level of burnout			P-value
		Low	Medium	High	
Long duties	Yes	12.2%	70.6%	17.3%	0.001
	No	27.3%	68.2%	4.5%	
Irregular working hours	Yes	13.4%	68.5%	18.1%	0.01
	No	20.2%	74.2%	5.6%	
Time away from home	Yes	14.6%	65.7%	19.7%	0.018
	No	16.1%	75.5%	8.4%	
Anti-social hours	Yes	12.5%	71.1%	16.4%	0.345
	No	17.8%	69.2%	13%	
Inflexible annual leave	Yes	9.3%	70.2%	20.5%	0.001
	No	20.6%	70%	9.4%	
Work conflicts	Yes	9%	70.1%	20.8%	0.001
	No	20.3%	70.1%	9.6%	
Unhealthy diet	Yes	11.8%	72.3%	16%	0.396
	No	17.3%	68.8%	13.9%	
Miscommunication at work	Yes	10.1%	67.2%	22.7%	0.003
	No	18.3%	71.8%	9.9%	
Changing nature of the industry	Yes	6.4%	70.5%	23.1%	0.006
	No	18.1%	70%	11.9%	
Sedentary nature of the job	Yes	8.2%	69.9%	21.9%	0.039
	No	17.3%	70.2%	12.5%	

## Discussion

The field of aviation has encountered an increase in challenges, and the demanding circumstances associated with being a professional pilot are merely an outcome of this situation [23]. Undoubtedly, completely eliminating stress from the professional lives of pilots is an unattainable goal. However, it is important to recognize that a high-stress environment does not necessarily have negative effects on individuals, as long as they have acquired effective coping mechanisms to manage it healthily. It is important to note that individuals employ either adaptive or maladaptive strategies to deal with stress, and substituting maladaptive coping mechanisms with more adaptive ones is a crucial aspect of therapeutic interventions and prognoses [24]. 

Our study findings unveiled a substantial prevalence of burnout among pilots, with 70.1% reporting a medium level of burnout. Upon comparing our study results, it was evident that 25.63% of pilots encountered work-related burnout, displaying a moderate or higher severity level [25]. Additionally, the prevalence of burnout within the Saudi aviation industry was reported to be 40.1% [26]. The dissimilarity in prevalence can be attributed to the differences in the populations surveyed in both studies. Our study exclusively targeted pilots, whereas the other study encompassed a broader spectrum of occupations within the aviation industry. Furthermore, our findings have unveiled significant correlations between burnout among pilots and several factors, specifically nationality and marital status. These findings suggest that personal factors play a role in the occurrence of burnout among pilots. In line with these findings, it is noteworthy that married pilots reported higher levels of burnout compared to their single counterparts [27]. This can potentially be attributed to the additional pressures outside of work and work-family conflicts that married pilots may encounter. Considering the nature of their profession, which often entails spending significant time away from home due to flight schedules, the challenges faced by married pilots can be further amplified. 

The impact of disrupted sleeping and eating patterns on pilots is indeed noteworthy. Due to the nature of their operations, pilots frequently experience significant disturbances in their daily routines. For instance, those involved in short-range flights may undergo a week of early shifts, commencing as early as 5 am, followed by a week of late shifts, concluding as late as 2 am. This irregular scheduling can greatly disrupt their natural circadian rhythm, resulting in disturbances in both sleeping and eating patterns [24]. In this study, a significant correlation was found between shorter sleeping duration and higher burnout levels highlighting the importance of sufficient rest and sleep for pilots' well-being and suggesting that addressing sleep-related issues could potentially reduce burnout levels. Pilots revealed a multitude of work-related stressors that were strongly linked to burnout. These stressors encompassed factors such as prolonged duty periods, unpredictable work hours, extended periods away from home, work conflicts, and instances of miscommunication within the workplace. Additionally, the evidence demonstrated that the three primary sources of work-related stress, ranked in descending order, were irregular working hours (72.07%), non-standard work hours (59.46%), and misalignment of values between management and pilots (54.95%). These findings underscore the significance of addressing these stressors to mitigate burnout among pilots [24].

Evidence has underscored the correlation between stress coping strategies and burnout, indicating that different coping strategies can predict the level of burnout [28]. Among pilots, rest and sleep, exercise, and relaxation behaviors were commonly reported coping strategies. While these strategies did not demonstrate statistical significance in reducing burnout levels, they remain important for pilots' overall well-being and should be encouraged as part of a comprehensive approach to managing stress and burnout. Undoubtedly, stress coping is a vital psychological concept that influences the relationship between stressors and behavioral outcomes, such as flying performance [29]. Regarding coping strategies, a strong emphasis has been placed on sleep and rest (29.28%), diet (26.47%), and exercise (13.61%), with comparatively less focus on relaxation methods (2.84%) [24]. Furthermore, a recent study highlighted the protective effect of mindfulness in mitigating burnout among civil pilots, highlighting the importance of mindfulness as a crucial factor for pilots to guard against burnout [28]. Furthermore, the importance of social support in moderating the relationship between perceived stress and mental health has been emphasized. Individuals who have stronger social support networks demonstrate improved capabilities to effectively manage and navigate stress, leading to better mental health outcomes as it acts as a protective mechanism, guarding against the negative impacts of stress and facilitating the adoption of effective coping strategies, ultimately promoting overall mental well-being [30].

This study has limitations, as the research sample only included male commercial pilots using a non-probability snowball sampling method, which may limit the generalizability of the findings to both genders and other occupations. Additionally, the study used a cross-sectional design, making it difficult to predict changes in pilots' burnout levels over time. Despite these limitations, the study provides valuable insights into burnout prevention and extends previous research. To our knowledge, it is the first of its kind conducted in the Kingdom of Saudi Arabia, enhancing our understanding of burnout in this specific context and providing a foundation for targeted interventions and strategies to address burnout among pilots in the Kingdom of Saudi Arabia. Nonetheless, the study adds to the knowledge base and serves as a valuable resource for future investigations and initiatives aimed at promoting the well-being and mental health of commercial pilots in the region.

## Conclusions

This study has shed light on the prevalence of burnout among pilots in different Saudi commercial airlines, with 70.1% of pilots experiencing moderate burnout levels. The study has identified several factors associated with burnout, including prolonged duty periods, unpredictable work hours, extended time away from home, work conflicts, and instances of miscommunication in the workplace. Furthermore, nationality, marital status, and shorter sleep duration were found to be associated with higher levels of burnout. These findings highlight the need to address work-related stressors and implement interventions to support the mental health and well-being of pilots. The study emphasizes the importance of promoting healthy coping strategies and developing a better understanding of individual resilience in the face of work-related challenges. By gaining insight into coping strategies, valuable lessons can be learned to enhance pilots' ability to withstand the pressures of their work environment. Further research and organizational efforts are necessary to effectively mitigate burnout and improve the overall quality of life for aviation professionals. By addressing these issues, the aviation industry can create a healthier and more supportive environment for pilots, ultimately benefiting both the individuals and the industry as a whole.
